# Effects of Puerariae Radix Extract on Endotoxin Receptors and TNF-**α** Expression Induced by Gut-Derived Endotoxin in Chronic Alcoholic Liver Injury

**DOI:** 10.1155/2012/234987

**Published:** 2012-10-21

**Authors:** Jing-Hua Peng, Tuan Cui, Zhao-Lin Sun, Fu Huang, Liang Chen, Lin Xu, Qin Feng, Yi-Yang Hu

**Affiliations:** ^1^Institute of Liver Diseases, Shuguang Hospital Affiliated to Shanghai University of Traditional Chinese Medicine, Shanghai 201203, China; ^2^Key Laboratory of Liver and Kidney Diseases, Ministry of Education, of Shanghai University of Traditional Chinese Medicine, Shanghai 201203, China; ^3^Shanghai Institute of Materia Medica, Chinese Academy of Sciences, Shanghai 201203, China; ^4^E-Institute of TCM Internal Medicine, Shanghai Municipal Education Commission, Shanghai 201203, China

## Abstract

Kudzu (*Pueraria lobata*) is one of the earliest medicinal plants used to treat alcohol abuse in traditional Chinese medicine for more than a millennium. However, little is known about its effects on chronic alcoholic liver injury. Therefore, the present study observed the effects of puerariae radix extract (RPE) on chronic alcoholic liver injury as well as Kupffer cells (KCs) activation to release tumor necrosis factor alpha (TNF-**α**) induced by gut-derived endotoxin in rats and macrophage cell line. RPE was observed to alleviate the pathological changes and lipids deposition in liver tissues as well as the serum alanine aminotransferase (ALT), aspartate aminotransferase (AST), and hepatic gamma-glutamyl transpeptidase (GGT) activity. Meanwhile, RPE inhibited KCs activation and subsequent hepatic TNF-**α** expression and downregulated the protein expression of endotoxin receptors, lipopolysaccharide binding protein (LBP), CD14, Toll-like receptor (TLR) 2, and TLR4 in chronic alcohol intake rats. Furthermore, an *in vitro* study showed that RPE inhibited the expression of TNF-**α** and endotoxin receptors, CD14 and TLR4, induced by LPS in RAW264.7 cells. In summary, this study demonstrated that RPE mitigated liver damage and lipid deposition induced by chronic alcohol intake in rats, as well as TNF-**α** release, protein expression of endotoxin receptors *in vivo* or *in vitro*.

## 1. Introduction

Alcohol abuse is a public issue and one of the major causes of liver disease worldwide. It covered large proportion of the deaths from liver disease not only in western world [1], but also in the eastern countries. Since 1980s the morbidity and mortality of alcoholic liver disease (ALD) has increased abruptly and presented a more serious trend in recent years in China [2].

ALD encompasses a spectrum of injury, ranging from simple steatosis, hepatitis, and cirrhosis. In alcoholic fatty liver, even the fatty drops disappear on abstinence and steatosis still increase the susceptibility of hepatocyte to further injury [3–7]. The continued ingestion of alcohol results in the subsequent steatohepatitis characterized with neutrophil infiltration, hepatocyte degeneration, ballooning, and oncotic necrosis [8, 9] that rarely recover to normal hepatic histology even with ethanol withdrawal [9]. Alcoholic steatohepatitis triggers the pathological progress to fibrosis and then cirrhosis, so as to this stage appears to represent a rate-limiting step in the progression of ALD [10, 11]. Therefore, blocking or reversing the early-phase histopathologic changes (steatosis and steatohepatitis) is the key strategy of ALD treatment.

**Figure 1:**
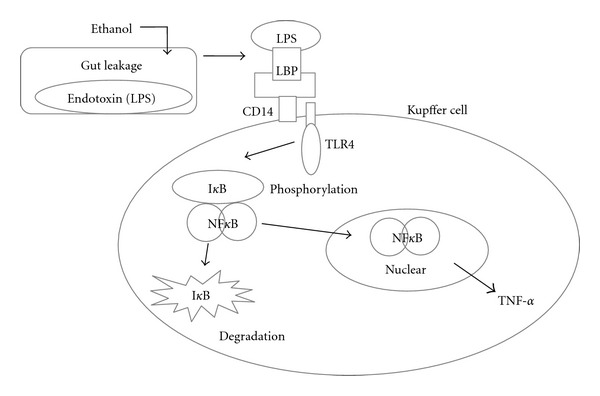
Mechanism of Kupffer cells activation releasing TNF-*α* to promote liver injury induced by gut-derived endotoxin in alcoholic liver disease.

Besides alcohol metabolism and oxidant stress, endotoxin, a toxic lipopolysaccharide (LPS) component of the gut Gram-negative bacteria, has also been revealed to be one of the classical mechanisms of alcoholic liver injury [12, 13]. As a result of increased intestinal permeability caused by alcohol, endotoxin releases into the circulation, transporting in complex of LPS and LPS-binding protein (LBP) and then binds to the CD14 receptor on hepatic Kupffer cells (KCs). The CD14-LBP-LPS complex interacts with toll-like receptor 4 (TLR4) to trigger a signaling cascade in KCs that activates nuclear factor *κ*B (NF*κ*B) and promotes the transcription of preinflammatory cytokines, especially tumor necrosis factor alpha (TNF-*α*) [14]. TNF-*α* can itself further increase gut permeability as well as oxidant stress and induces apoptosis and production of other cytokines, perpetuating, and progressing liver injury [15] (Figure 1).

Kudzu (*Pueraria lobata*) is one of the earliest medicinal plants used to treat alcohol abuse in traditional Chinese medicine for more than a millennium. It has been revealed that antidipsotropic isoflavones isolated from radix puerariae, including puerarin, daidzin, and daidzein, suppressed the ethanol intake of rodents, abolished the development of alcohol withdrawal symptoms [16–23], increased the antioxidant enzymes (such as Cu/Zn SOD and catalase [CAT]), and mitigated the hepatic oxidant injury in ethanol-treated rats [24]. But few investigations on the mechanisms of the radix puerariae extract (RPE) antialcoholic liver injury were conducted comprehensively. 

In the present study, we investigated the effects of RPE on chronic alcoholic liver injury in rats as well as the expression of endotoxin receptors and TNF-*α* induced by gut-derived endotoxin *in vivo* and *in vitro*. 

## 2. Materials and Methods 

### 2.1. Preparation of PRE

Radix puerariae, the root of *Pueraria lobata* (Willd) Ohwi (Shanghai Leiyunshang Pharmaceutical Co. Ltd, Shanghai, China) was powered and extracted with 70% alcohol for two hours, three times, concentrated with the vacuum rotary evaporator and freeze-dried. The dried powder was then dissolve in distilled water, filtered, and subjected to a macroporous resin D101 column (11.5 cm × 85.5 cm), eluting with distilled water and 70% ethanol. The 70% ethanol eluting fluid was dried with a rotary evaporator and then stored desiccated in dark at 4°C. 3.89 g of RPE was obtained from 100 g of the dried Radix puerariae.

### 2.2. Analysis of RPE

The standard constituents used for the quantitative analysis of RPE were puerarin, daidzin, and daidzein, which are currently recommended for quality control of radix puerariae. These standards were purchased from Shanghai Win herb Medical Scientific & Technology Development Co. Ltd (Shanghai, China).

The standard compounds and RPE were dissolved in 30% ethanol, filtered through 0.2 *μ*m nylon filters, and analyzed by HPLC procedures. The HPLC apparatus (Agilent 1200 Series, USA) was employed for analysis. The chromatographic separation was carried out using a mixture of acetonitrile (A) and water containing 0.02 mol/ul phosphoric acid (B), and the mobile phase was conducted as Table 1 demonstrating. The column eluent was monitored at UV 250 nm. The chromatography was performed at room temperature with a flow rate of 1.0 mL/min, and 10 *μ*L volume was analyzed.

**Figure 2:**
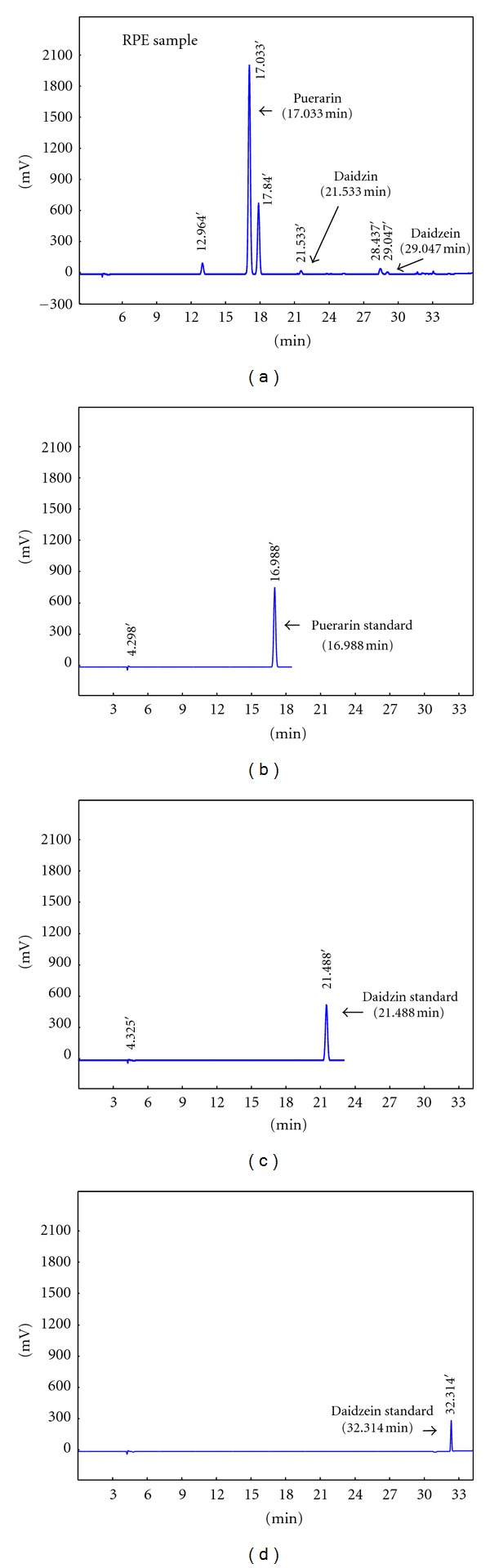
HPLC chromatograms of the RPE and the standard compounds: (a) RPE, (b) puerarin standard (16.988 min), (c) Daidzin standard (21.488 min), and (d) daidzein (32.314 min).

Analysis showed that 100 mg RPE contained 49.5 mg puerarin, 1.2 mg daidzin, and 0.15 mg daidzein, indicating that puerarin was the major constituent in RPE (Table 1, Figure 2).

### 2.3. Animals and Treatments

Male Sprague-Dawley (SD) rats (160 ± 10 g) were obtained from Shanghai laboratory animal center of Chinese academy of sciences (Shanghai, China) and acclimatized for 7 days after delivery. All rats were maintained on a reverse 12 h light : 12 h dark cycle. Animal handling and procedures were performed according to international guidelines for the use and care of laboratory animals. The experimental protocol was approved by the local ethics committee.

Rats were divided into four groups: control (*n* = 10), ethanol (*n* = 10), ethanol plus high-dosage RPE (240 mg/kg·d, *n* = 10), and ethanol plus low-dosage RPE (120 mg/kg·d, *n* = 10) group. Lieber-DeCarli control and ethanol liquid diet were prepared according to the prescription of Lieber-DeCarli diet [25] as described in the previous research [26]. Rats in control group ingested Lieber-DeCarli control liquid diet and rats in ethanol or drug-administrated group ingested Lieber-DeCarli alcohol liquid diet. Ethanol provided 36% energy in the alcohol liquid diet, which was substituted by dextrin-maltose in the control liquid diet. One liter liquid diet contains 1000 Kcal energy. Reagents in the Liber-DeCarli formula were purchased from Sinopharm Chemical Reagent Co., Ltd (Shanghai, China). Rats were single-cage rearing and liquid diet was intake freely without additional water or chow for eight weeks. At the beginning of the third week, rats in ethanol plus high- and low-dosage RPE groups were administrated with RPE by gavage in 240 mg/kg·d and 120 mg/kg·d, respectively, the others with equal volume of sterile water. At the end of the eighth week, rats were anaesthetized with nembutal (45 mg/kg, i.p.); liver tissue and serum were collected and stored in −80°C for histological, biochemical, and Western blot analysis. Two milliliter blood from portal vein was collected in pyrogen-free and heparin-pretreated tube for endotoxin detection. 

### 2.4. Cell Culture and Treatment

The murine macrophage cell line, RAW264.7 cells, (Shanghai Institutes for Biological Sciences, Chinese Academy of Sciences, Shanghai, China) were cultured in RPMI-1640 (GIBCO Invitrogen Corporation, CA, USA) containing 10% fetal bovine serum (FBS) (GIBCO) in 5% CO_2_ incubator (Heraeus Holding GmbH, Germany) at 37°C. Cultured for 24–48 h, approximately 70% confluence, RAW264.7 cells were divided into five groups: control, LPS, 0.84 mg/L RPE (containing 1 nM puerarin) plus LPS, and 8.4 mg/L (containing 10 nM puerarin) RPE plus LPS group. RPE was dissolved in dimethyl sulfoxide (DMSO) and diluted with RPMI-1640 (final concentration of DMSO was 0.005‰). After preincubated with RPE for 4 h, the cells were treated with LPS (Escherichia coli 0111 : B4, Sigma-Aldrich Co., USA) 0.1 *μ*g/mL for 2 h without rinse. And then the supernatant and cellular proteins were collected for assay.

### 2.5. LDH Secretion Test in RAW264.7

RAW264.7 cells were cultured with 10% FBS RPMI-1640 (control), 84 mg/L RPE (containing 100 nM puerarin) plus 10% FBS RPMI-1640, 840 mg/L RPE (containing 1 *μ*M puerarin) plus 10% FBS RPMI-1640, and 8.4 g/L RPE (containing 10 *μ*M puerarin) plus 10% FBS RPMI-1640, respectively. After 6 hours, the activity of lactate dehydrogenase (LDH) in the supernatant was detected. The LDH activity was corrected with the cellular protein concentration in every dish and expressed as U/pg proteins. 

### 2.6. Histological Examination

Liver tissue was formalin-fixed and embedded in paraffin. Sections (4 *μ*m thick) were stained with hematoxylin-eosin (H.E.) (Nanjing Jiancheng Bioengineering institute, Nanjing, China) and examined under light microscope (Olympus Medical Systems Corp., Tokyo, Japan).

Frozen hepatic tissue (7 *μ*m thick) embedded in optimum cutting temperature (OCT) compound (Sakura Finetek USA, Inc., CA, USA) was stained with oil red O (Sinopharm Chemical Reagent Co., Ltd, Shanghai, China) for hepatic lipid observation (Olympus Medical Systems Corp., Tokyo, Japan).

### 2.7. Serum ALT and AST Assay

Activity of alanine aminotransferase (ALT), aspartate aminotransferase (AST) in serum was determined with the corresponding biochemical assay kits (Nanjing Jiancheng Bioengineering institute, Nanjing, China).

### 2.8. Hepatic GGT Assay

Liver tissue (100 mg) was homogenized in 1 mL 0.9% NaCl. The homogenate was centrifuged at 1,000 g, 4°C for 15 min, the supernatant was removed into clean tubes and centrifuging at 3,000 g for 10 min, avoiding the upper adipose and removing the transparent for gamma-glutamyl transpeptidase (GGT) assay (Nanjing Jiancheng Bioengineering institute, Nanjing, China).

### 2.9. Hepatic TG Assay

Liver tissue (200 mg) was homogenized in 3 mL ethanol-acetone mixture (1 : 1 in volume). The total hepatic triglyceride (TG) extracted in the medium at 4°C overnight and then, centrifuged at 1,000 g, 4°C for 20 min, the supernatant was removed for TG assay with TG analysis kits (Dongou Bioengineering Co. Ltd, Zhejiang, China).

### 2.10. Endotoxin Assay in Plasma from Portal Vein

Blood collected from portal vein was centrifuged at 500 g, 4°C for 15 min, plasma was removed immediately for analysis, according to the instruction of Pyrochrome limulus amebocyte lysate (LAL) kit (Cat NO. C1500, Associates of Cape Cod, Inc., TX, USA).

### 2.11. Immunohistochemical Assessment of Hepatic CD68

As discribed in previous study [27], 4 *μ*m thick paraffin sections were used for immunohistochemical assessment. Briefly, after endogenous peroxidase blockage and bovine serum albumin blockage, the samples were incubated at 4°C overnight, with a 1 : 100 dilution of anti-CD68 primary antibody (monoclonal anti-rat CD68, AbD Serotec, NC, USA). Following the processing of the samples incubated with a 1 : 250 dilution of horseradish-peroxidase-(HRP-) linked goat anti-mouse IgG (sc-2031, Santa Cruz Biotechnology Inc. Santa Cruz, CA) for 1 h at 37°C, diamino benzidine (DAB) was applied as a chromogen and hematoxylin was used for floor staining.

### 2.12. Measurement of TNF-*α* Level by ELISA

Liver TNF-*α* was isolated as described in previous research [28], liver pieces (1.0 g) were minced thoroughly in ice-cold radioimmunoprecipitation assay buffer (150 mM NaCl, 5 mM EDTA, 50 mM Tris base, 0.3% Triton X-100, 0.03% sodium dodecyl sulfate, 0.3% Na-deoxycholate, and 1% protease inhibitor cocktail, pH 7.4) and lysised on ice for 30 min. The liver tissue extracts were then centrifuged at 15,000 g for 20 min at 4°C. Removing the supernatants to clean tubes and centrifuging again at 15,000 g for 20 min at 4°C, the supernatants of this spin were then used for enzyme-linked immunosorbent assay (ELISA). TNF-*α* in both hepatic tissue and the culture supernate of RAW364.7 cell was determined by a commercially available ELISA kit (Cat. NO. KRC3012, Invitrogen Corporation, Camarillo, CA, USA) according to the manufacturer's instruction. The result was corrected by protein concentration (bicinchoninic acid [BCA] protein concentration assay kit, Beyotime Inst. Biotechnology, Jiangsu, China). 

### 2.13. Determination of CD68 and Endotoxin Receptors in Liver Tissue by Western Blot

As described previously [27, 29], total proteins in liver tissue and cell layers were extracted, analyzed with BCA protein concentration assay kit (Beyotime Inst. Biotechnology, Jiangsu, China). 

Sample protein was separated by electrophoresis in 10% SDS-PAGE separating gel with Bio-Rad electrophoresis system (BioRad Laboratories, Hercules, CA, USA). The primary antibodies (mouse anti-rat glyceraldehydes-3-phosphate dehydrogenase, GAPDH antibody, 1 : 5000 dilution, KANGCHEN Bio-Tech Inc., Shanghai, China; mouse anti-rat CD68 antibody, 1 : 100 dilution, AbD Serotec, NC, USA; mouse anti-rat CD14 antibody, 1 : 200 dilution, Santa Cruz biotechnology Inc., CA, USA; goat anti-rat LBP antibody, 1 : 100 dilution, Santa Cruz biotechnology Inc., CA, USA; rabbit anti-rat TLR2 antibody, 1 : 2000 dilution, Epitomics, Inc. CA, USA; rabbit anti-rat TLR4 antibody, 1 : 200 dilution, Santa Cruz biotechnology Inc., CA, USA) were incubated at 4°C overnight. The corresponding HRP-conjugated secondary antibodies (goat anti-mouse IgG, goat anti rabbit-IgG peroxidase linked antibody, 1 : 5000 dilution, Santa Cruz Biotechnology Inc., CA, USA; rabbit anti-goat-IgG, peroxidase linked antibody, 1 : 5000 dilution, Jackson ImmunoResearch Laboratories Inc., PA, USA) were incubated at room temperature for 1 h. The ECL kit (Pierce Biotechnology Inc., Rockford, USA) and the Furi FR-980 image analysis system (Shanghai Furi Co., Shanghai, China) were employed for revealing and quantitative analysis of the blots. GAPDH protein was used as the internal control.

### 2.14. Statistical Analysis

All results were expressed as mean ± SD. The data were analyzed using a one-way analysis of variance (ANOVA) followed by the least significant difference (LSD) post hoc test. *T*-test is employed for the comparison of two parameters. Differences were considered statistically significant if the *P* value < 0.05.

## 3. Results

### 3.1. Liquid Diets Intake, Body Weight, and Liver/Body Weight Ratio

At the end of the eighth week, the total volume of liquid diets and the average body weight was no significantly different in various groups (*P* > 0.05). The liver/body weight ratio of rats fed with ethanol liquid diets increased significantly compared with that of rats in control group (*P* < 0.01), and there is no significance statistically between RPE groups and ethanol group (Table 2).

### 3.2. Effects of RPE on Live Injury Induced by Chronic Alcohol Intake

**Figure 3:**
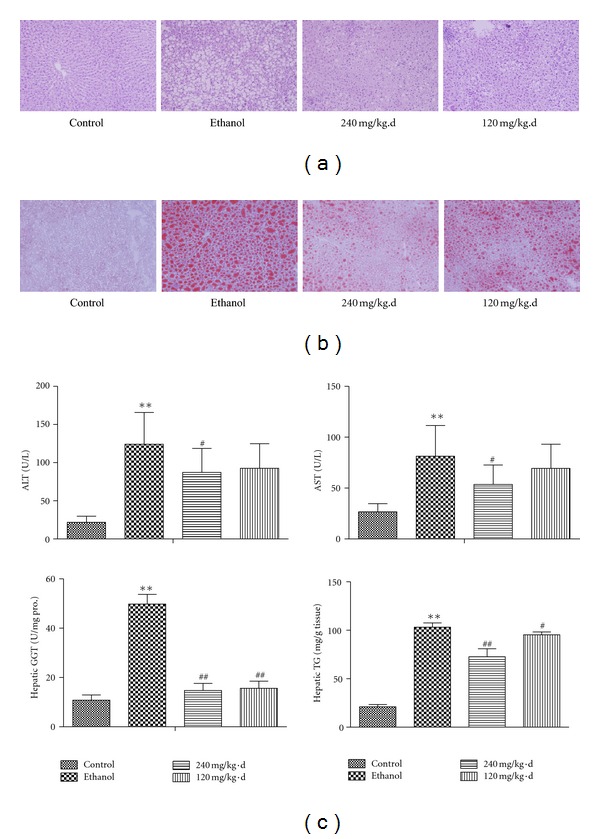
Effects of RPE on liver injury and lipid deposition induced by Lieber-DeCarli diet. (a) Histological observation on the H.E. sections (original magnification, ×200). (b) Hepatic lipid droplets observation on red O staining sections (original magnification, ×200). (c) Serum ALT, AST, hepatic GGT, and TG variation. Values represent the mean ± SD of 10 rats. *P < 0.05, versus control; ^#^ *P* < 0.05, versus ethanol; **P < 0.01, versus control, ^##^ *P* < 0.01, versus ethanol.

Chronic alcoholic liver injury was examined by biomarkers of liver damage and histological changes in liver tissue. The serum ALT and AST activities significantly increased in ethanol group compared with that in the control (ALT: 124.02 ± 41.68 U/L versus 21.81 ± 7.90 U/L, *P* < 0.01; AST: 81.25 ± 30.16 U/L versus 26.70 ± 7.99 U/L, *P* < 0.01). The serum ALT of rats in administration with high-dosage RPE group was obviously decreased (87.26 ± 31.37 U/L versus 124.02 ± 41.68 U/L, *P* < 0.05). The activity of serum AST in high-dosage RPE group demonstrated similar trends (53.51 ± 19.16 U/L versus 81.25 ± 30.16 U/L, *P* < 0.05). Decrease of these two biomarkers in low-dosage RPE group did not appear statistically significance (ALT: 92.58 ± 32.14 U/L versus 124.02 ± 41.68 U/L, *P* > 0.05; AST: 69.33 ± 23.72 U/L versus 81.25 ± 30.16 U/L, *P* > 0.05). On the other hand, hepatic GGT activity increased after ethanol liquid diet intake obviously (49.75 ± 3.97 U/*μ*g pro. versus 10.83 ± 2.11 U/*μ*g pro., *P* < 0.01), and after RPE administration, hepatic GGT activity decreased significantly (14.71 ± 2.90 U/*μ*g pro. versus 49.75 ± 3.97 U/*μ*g pro., *P* < 0.01; 15.63 ± 2.19 U/*μ*g pro. versus 49.75 ± 3.97 U/*μ*g pro., *P* < 0.01) (Figure 3(c)).

After chronic alcohol intake for eight weeks, the macrovesicular steatosis was observed in the most region of lobule, predominantly in centrilobular regions. Lipid vacuoles occupied much of the hepatocyte cytoplasm, and the nucleus and other organelles were pushed to the periphery of the cell. Some hepatocytes appeared bloated, with a wispy, rarefied cytoplasm. The inflammatory cells, such as neutrophil and lymphocytes, scattered located in the pericellular region. RPE of high-dosage or low-dosage treatment alleviated the pathological changes mentioned above (Figure 3(a)).

### 3.3. Effects of RPE on Hepatic Lipid Deposition

The concentration of TG in liver tissue and oil red O staining of liver tissue section were employed to demonstrate the hepatic lipid deposition.

The total TG extracted by liver tissue homogenization was tested. As expected, chronic alcohol exposure elicited almost 5-fold increase in hepatic TG levels as compared with control animals (103.53 ± 13.59 mg/g liver tissue versus 22.39 ± 9.19 mg/g liver tissue, *P* < 0.01). The levels of hepatic TG decreased remarkably in RPE-treated animals (high-dosage RPE, 72.90 ± 24.15 mg/g liver tissue versus 103.53 ± 13.59 mg/g liver tissue, *P* < 0.01; low-dosage RPE, 95.64 ± 9.41 mg/g liver tissue versus 103.53 ± 13.59 mg/g liver tissue, *P* < 0.05) (Figure 3(c)). 

Oil red O staining slides showed that in the hepatocytes cytoplasm of chronic drinking rats, there were large droplets colored with oil red O, which were widespread distributed in the hepatic lobule, indicating severe steatosis in alcohol intake animals. In animals treated with RPE in high or low-dosage, the droplets were smaller and limited, which indicated that hepatic steatosis was mitigated with RPE administration (Figure 3(b)).

### 3.4. Endotoxin Level in the Portal Vein and Hepatic TNF-*α* Concentration

**Figure 4:**
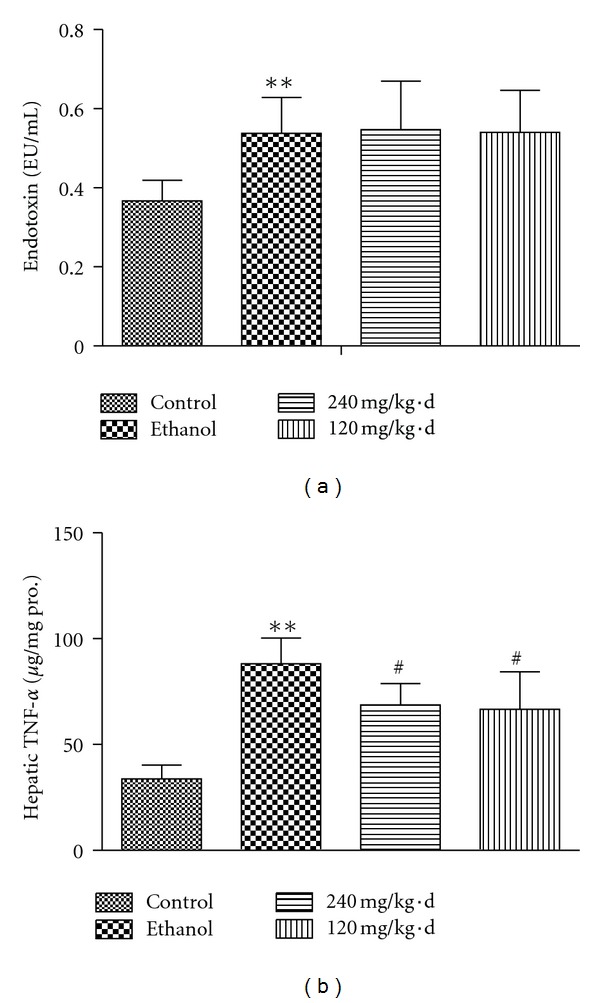
Effects of RPE on endotoxin in the portal vein and hepatic TNF-*α* concentration. (a) Endotoxin level in portal vein, (b) TNF-*α* concentration in liver tissue. Values represent the mean ± SD of 10 rats. **P < 0.01, versus control, ^#^ *P* < 0.05, versus ethanol.

Endotoxin level in the portal vein reflects the situation of endotoxin leakage from gut and indicates the permeability of intestinal wall, indirectly. In the present study, the endotoxin level in the portal vein was found increasingly remarkably after long-term ethanol intake (0.538 ± 0.09 EU/mL versus 0.367 ± 0.052 EU/mL, *P* < 0.05); however, not varying after RPE administration (high-dosage RPE versus ethanol, 0.547 ± 0.12 EU/mL versus 0.538 ± 0.09 EU/mL, *P* > 0.05, low-dosage RPE versus ethanol, 0.54 ± 0.11 EU/mL versus 0.538 ± 0.09 EU/mL, *P* > 0.05) (Figure 4(a)). This result suggested that chronic alcohol exposure promoted endotoxin leakage from intestine, which was not inhibited by RPE administration.

After entering circulation, the gut-derived endotoxin activates Kupffer cells to release preinflammatory factors, such as TNF-*α*, promoting liver injury. In the present study, hepatic TNF-*α* was detected and it was observed that hepatic TNF-*α* concentration increased significantly in ethanol group (88.18 ± 12.08 *μ*g/mg pro. versus 33.76 ± 6.50 *μ*g/mg pro., *P* < 0.05), and with RPE administration, it decreased obviously (68.64 ± 10.19 *μ*g/mg pro. versus 88.18 ± 12.08 *μ*g/mg pro., *P* < 0.05; 66.70 ± 17.66 *μ*g/mg pro. versus 88.18 ± 12.08 *μ*g/mg pro., *P* < 0.05), which is inconsistent with the RPE effects on endotoxin level in portal vein (Figure 4(b)). 

### 3.5. Effects of RPE on Kupffer Cells Activation and Protein Expression of Endotoxin Receptors in Liver

Since no obvious inhibitory effect of RPE on endotoxin leakage from gut was observed, the effect of RPE on KCs activation and endotoxin receptors protein expression were then detected for investigating the mechanism of RPE block TNF-*α* release.

**Figure 5:**
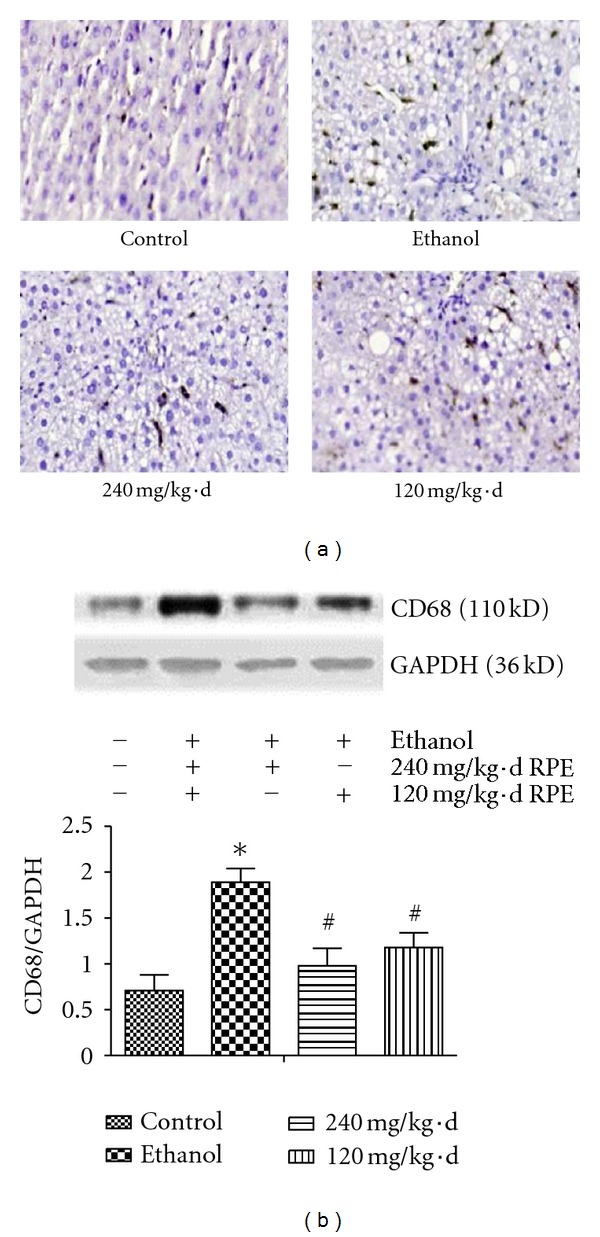
Effects of RPE on Kupffer cells activation in liver. (a) Hepatic CD68 expression detected with immunohistology (original magnification, ×400), (b) Hepatic CD68 expression detected with western-blot. Values represent the mean ± SD of three independent experiments. *P < 0.05, versus control; ^#^ *P* < 0.05, versus ethanol.

CD68/macrosialin, a transmembrane protein expressed by activated tissue macrophages [30], was detected as a marker of the activated KCs. Immunohistological assay showed that few CD68-positive staining was observed in the hepatic sinusoidal of control rats (Figure 5(a)). The positive staining in hepatic tissue was stronger and the areas were enlarged in the livers of ethanol-diet raised rats (Figure 5(a)). While CD68 expression decreased in the liver sections of RPE high- or low-dosage administrated rats, respectively (Figure 5(a)), these results were confirmed by western-blotting assay (Figure 5(b)).

**Figure 6:**
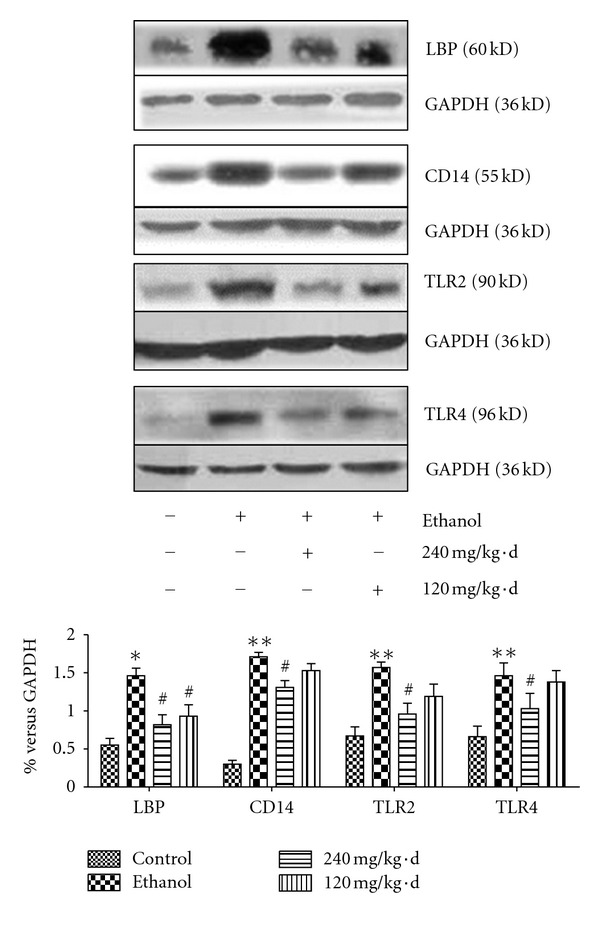
Effects of RPE on protein expression of endotoxin receptors in liver. RPE inhibited protein of endotoxin receptors, LBP, CD14, TLR4, and TLR2 (western-blot). Values represent the mean ± SD of three independent experiments. *P < 0.05, versus control; **P < 0.01, versus control; ^#^ *P* < 0.05, versus ethanol.

LBP, the essential protein for LPS-transferring in the circulation, is predominantly produced by liver [31], which binds to LPS of Gram-negative bacteria with high affinity to form the LPS-LBP complex and transfer of LPS to the surface receptors on target cells (KCs), such as membrane CD14. The pattern recognition receptors, TLRs, recognize the signal of LPS delivered by LBP and CD14 and activate the downstream cascades. As been disclosed in protein expression assay, in ethanol-diet group, the protein expression of endotoxin receptors in liver tissue, such as LBP, CD14, TLR4, and TLR2, were increased remarkably compared to that in control group. While administrated with RPE, the protein expression of endotoxin receptors were downregulated significantly, especially, in the high-dosage group (Figure 6). 

The results obtained from *in vivo* study suggested that RPE mitigated alcoholic liver injury and hepatic lipid deposition induced by Lieber-DeCarli diet in rats as well as TNF-*α* release, protein expression of endotoxin receptors, and KCs activation in liver. Since the endotoxin level in portal vein did not vary between ethanol and RPE groups, RPE was not indicated probably to inhibit endotoxin leakage from intestinal induced by long-term ethanol exposure. To confirm the direct effects of RPE on TNF-*α* and endotoxin receptors expression in KCs induced by LPS, the macrophage cell line, RAW264.7, was employed in the subsequent experiments *in vitro*. 

### 3.6. Activity of LDH in RAW264.7 Supernatant Cultured with Different Concentrations of RPE

The LDH test in RAW264.7 was employed to determine the security range of RPE dosage on cells *in vitro*. Increase of LDH indicates cell injury and the toxicity of drug.

The toxicity of RPE concentration range of 84 mg/L (containing 100 nM puerarin), 840 mg/L (containing 1 *μ*M puerarin), and 8.4 g/L (containing 10 *μ*M puerarin) on RAW264.7 was examined by LDH activity assay in the culture supernatant. After cultured for 6 h, as dosage of RPE increased (84 mg/L, 840 mg/L, 8.4 g/L), LDH activity did not increase comparing to that in control and even decreased in the 84 mg/L RPE group. That indicated that RAW264.7 cells cultured with 8.4 g/L RPE or lower concentration was secure (Table 3).

### 3.7. Effect of RPE on TNF-*α* Secretion in RAW264.7 Cell Culture Supernatant Induced by LPS * In Vitro*

**Figure 7:**
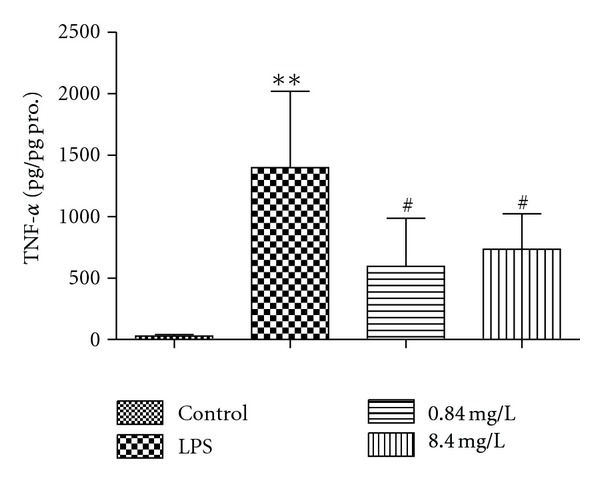
Effect of RPE on TNF-*α* secretion in RAW264.7 cell culture supernatant induced by LPS. RPE inhibited TNF-*α* secretion induced by LPS *in vitro*. Values represent the mean ± SD of three independent experiments. **P < 0.01, versus control; ^#^ *P* < 0.05, versus LPS.

The results showed that TNF-*α* secretion in the supernatant increased significantly (LPS versus control, 1397 ± 620 pg/pg pro. versus 29 ± 11 pg/pg pro., *P* < 0.01). Treatment with 0.84 mg/L (containing 1 nM puerarin) and 8.4 mg/L (containing 10 nM puerarin) RPE (pretreated for 4 h and then coincubated with LPS for 2 h) reduced the TNF-*α* secretion induced by LPS (0.84 mg/L RPE versus LPS, 594 ± 391 pg/pg pro. versus 1397 ± 620 pg/pg pro., *P* < 0.01; 8.4 mg/L RPE versus LPS, 733 ± 288 pg/pg pro. versus 1397 ± 620 pg/pg pro., *P* < 0.01). No dosage-dependent effects were observed (Figure 7). 

### 3.8. Effect of RPE on Endotoxin Receptors Protein Expression Induced by LPS * In Vitro*

**Figure 8:**
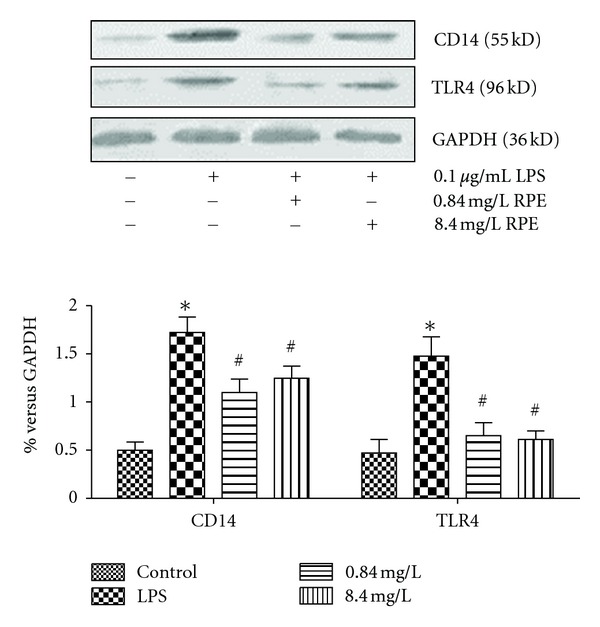
Effect of RPE on endotoxin receptors protein expression induced by LPS *in vitro*. RPE inhibited CD14 and TLR4 protein expression significantly *in vitro* (western-blot). Values represent the mean ± SD of three independent experiments. *P < 0.05, versus control; ^#^ *P* < 0.05, versus LPS.

The protein expression of endotoxin receptors, CD14 and TLR4 in RAW264.7 cells, was upregulated significantly after stimulated by LPS. Similarly, with treatment by 0.84 mg/L and 8.4 mg/L RPE (pretreated for 4 h and then coincubated with LPS for 2 h), CD14 and TLR4 protein expressions were downregulated without dosage-dependent effect (Figure 8).

## 4. Discussion

Kudzu (*Pueraria lobata*) has been used in China as an anti-inebriation agent for centuries. Puerarin, daidzin, and daidzein have been identified as the three major isoflavonoid compounds in RPE. Previous researches mostly focused on the antidipsotropic effect of isoflavonoids in RPE. The most impressive belongs to the series of studies on daidzin as a selective inhibitor of aldehyde dehydrogenase (ALDH-2) [32–35], and the artificial synthesis analogue with therapeutic potential to reduce ethanol intake and relapse abstinent alcoholics has been developed [36]. On the other hand, puerarin is found to be protective against acute alcoholic liver injury and alcoholism-related disorders by inhibiting the oxidative stress [37, 38] as well as reversed liver fibrosis induced by alcohol compound with carbon tetrachloride by promoting apoptosis of activated hepatic stellate cells (HSCs) [39]. The effect of RPE on chronic alcoholic liver injury and the mechanisms were rarely reported. 

In the present study, RPE was observed to inhibit chronic alcoholic liver injury. The histological examination and the biomarkers of liver damage, serum ALT, AST, and hepatic GGT, revealed that RPE alleviated the liver injury induced by Lieber-DeCarli liquid diet. The hepatic TG concentration test suggested that RPE decreased the hepatic lipid deposition induced by chronic alcohol intake, which was also observed in the oil red staining sections. 

The role of gut-derived LPS in the pathogenesis of ALD has been widely demonstrated. LPS is the primary endogenous endotoxin of gram-negative bacteria [12]. Long-term ethanol exposure leads to bacterial overgrowth in the gut, disruption of intestinal barrier function, and increase in permeability to endotoxin and bacteria. In response to the endotoxin, KCs produce proinflammatory cytokines, such as TNF-*α*, and cause the surrounding parenchymal cells damage. In the present study, chronic alcohol diet intake caused a significant increase of endotoxin in the portal vein and proinflammatory cytokine, TNF-*α*, secretion in liver tissue. Simultaneously, the protein expression of hepatic CD68 was upregulated remarkably, indicating KCs activation induced by chronic alcohol diet. However, with the administration of RPE, the level of endotoxin in the portal vein did not vary significantly comparing to that in ethanol group, indicating that RPE did not inhibit the endotoxin leakage from intestine induced by alcohol. On the other hand, the TNF-*α* expression and the KCs activation were all inhibited, which supported RPE blocking proinflammatory cytokine secretion by KCs in chronic liver injury. These results suggested that RPE inhibiting proinflammatory cytokine released by KCs probably did not through decrease in endotoxin leakage from gut induced by alcohol and effects of RPE on endotoxin receptors were then evaluated *in vivo* and *in vitro*. 

The signal of endotoxin delivery depends on the endotoxin receptors. LBP is produced mostly by hepatocytes and secreted into the bloodstream, where it binds with high affinity to the lipid A portion of LPS and catalyses the transfer of individual LPS molecules to cell surface receptors, such as membrane CD14 (mCD14), forming a monomeric LPS-CD14 complex. With LBP presence, the concentration of LPS sufficient for cellular activation is decreased significantly [40]. With the stimulation of proinflammatory cytokines, LBP is constitutively synthesized in hepatocytes [41], which is consistent with the result obtained in the present study, in the rats by in taking alcohol diet from long-term, the protein expression of hepatic LBP was upregulated markedly.

Molecular CD14 anchors on the membrane of peripheral or liver resident microphage (KCs) through glycosylphosphatidylinositol (GPI). LPS-CD14 complex activated cells through TLR4 [42]. It has been clearly established that TLR4 is the specific receptor of LPS from Gram-negative bacteria [42–44]. Signaling through TLR4 requires myeloid differentiated protein-2 (MD-2), a secreted protein that is closely associated with the extracellular domain of TLR4 [45]. In the present research, the protein expression of CD14 and TLR4 in liver tissue was found to be upregulated by chronic alcohol diet intake.

The downstream of TLR4, an endotoxin signal, then occurs through the interleukin-1(IL-1)-receptor pathway, which is MyD88 dependent, or alternatively via a MyD88-independent pathway [46]. Ultimately, the endotoxin signal activates NF*κ*B translocation into the nucleus where it induces proinflammatory gene expression, such as TNF-*α*, inducing liver injury. Therefore, the endotoxin receptors are essential for LPS signal delivery and the ultimate proinflammatory gene expression. In the present study, LBP, CD14, and TLR4 expression in liver tissue were all downregulated by RPE treatment comparing to that in ethanol group, which were consistent with its effect on hepatic TNF-*α*. Therefore, data from study *in vivo* indicated RPE inhibited hepatic TNF-*α* release induced by chronic alcohol intake probably via downregulating the expression of endotoxin receptors instead of endotoxin leakage from gut.

To observe the effect of RPE on endotoxin receptors and TNF-*α* expression in macrophage cells induced by LPS directly, the macrophage cell line RAW264.7 was employed in the present study. The results of experiments *in vitro* revealed that RPE inhibited the TNF-*α* expression induced by LPS as well as the protein expression of endotoxin receptors, CD14 and TLR4 in RAW264.7 cells, which confirmed the results obtained from study *in vivo*.

In addition, TLR2 appears to primarily respond to Gram-positive bacteria-derived lipoteichoic acid (LTA), peptidoglycan (PGN), and mycobacterial lipoarabinomannan [46]. However, MD-2 enables TLR2 to respond to the LPS and enhances TLR2-mediated responses to both Gram-negative bacteria and their LPS [47]. On the other hand, the cytokines induced by LPS, such as IL-1*β* or TNF-*α*, upregulate TLR2 mRNA expression in rat hepatocyte *in vivo* and *in vitro* [48]. In the present research, the protein expression of TLR2 was found remarkably upregulated in the liver tissue of alcohol-diet raised rats, but not in the LPS stimulated macrophage cell line RAW264.7, which is consistent with our previous studies [27]. Similarly, RPE treatment also downregulated the TLR2 protein expression in rats with chronic alcoholic intake.

## 5. Conclusion

In conclusion, the present study confirmed that RPE mitigated liver damage and lipid deposition induced by chronic alcohol intake as well as TNF-*α* release, protein expression of endotoxin receptors *in vivo* and *in vitro*.

## Figures and Tables

**Table 1:** HPLC mobile phase gradient.

Time (min)	Moble A (%)	Mobile B (%)
0.00	15	85
25.00	35	65
30	100	0
35	100	0

**Table 2:** Liquid diets intake, body weight, and liver/body weight ratio.

Group (*n*)	Volume of liquid diet intake (mL)	Body weight (g)	Liver/body weight ratio (g/kg body weight)
Control (10)	2726 ± 75	262 ± 28	20.06 ± 2.99
Ethanol (10)	2707 ± 218	257 ± 50	28.07 ± 5.70**
240 mg/kg·d RPE (10)	2723 ± 166	269 ± 22	31.17 ± 4.09
120 mg/kg·d RPE (10)	2726 ± 141	276 ± 24	29.45 ± 4.61

**P < 0.01, versus control.

**Table 3:** Activity of LDH in RAW264.7 supernatant cultured with different concentrations of RPE.

Group	LDH (U/pg proteins)
Control	14.61 ± 3.06
84 mg/L RPE	8.69 ± 4.06**
840 mg/L RPE	11.98 ± 6.57
8.4 g/L RPE	12.34 ± 4.26

**P < 0.01, versus control.
